# Usefulness of clinical observations and blood chemistry values for predicting clinical outcomes in dairy goats with pregnancy toxaemia

**DOI:** 10.1186/s13620-016-0075-4

**Published:** 2016-10-24

**Authors:** Miguel S. Lima, Júlia M. Silveira, Nuno Carolino, Luis P. Lamas, Rita A. Pascoal, Charles A. Hjerpe

**Affiliations:** 1 Faculdade de Medicina Veterinária, Centro de Investigação Interdisciplinar em Sanidade Animal (CIISA), DC, Universidade de Lisboa, Polo Universitário da Ajuda, Lisbon, 1300-477 Portugal; 2 INIAV, EUVG, CIISA, Lisbon, Portugal; 3 Barão e Barão Lda, Coutada Velha, Benavente, 2130-010 Portugal; 4 School of Veterinary Medicine, University of California, Davis, CA 95616 USA

**Keywords:** Pregnancy toxaemia, Goats, Clinical signs, Metabolic acidosis, Hypokalaemia, Prognosis

## Abstract

**Background:**

Pregnancy toxaemia (PT) is a disease that affects pregnant goats during their last month of gestation and is characterized by a high case fatality rate. This study involved 32 does maintained on a commercial dairy goat farm that were diagnosed with PT. A physical examination was performed on and haematology parameters obtained from each doe, at the time of diagnosis. The data from the 24 PT goats that died was compared with the corresponding data from the 8 PT goats that survived.

**Results:**

Polypnea, swollen limbs, anorexia with absence of ruminal motility, recumbency, nervous signs and drooping ears were the most frequently observed clinical manifestations. Nineteen out of 21 recumbent goats died. Sixteen out of 17 goats with anorexia and absence of ruminal motility died. Mean beta-hydroxybutyric acid (BHBA) values in the goats that died were not significantly different from those in goats that survived. The blood values for pH and pCO_2_ (*p* < 0.005) as well as for HCO_3_^−^, BE and K^+^ (*p* < 0.001) were significantly lower in the goats that died than in those that survived.

**Conclusions:**

The clinical signs most indicative of a poor prognosis are anorexia with absence of ruminal motility and recumbency. Among the blood parameters to be considered, hypokalaemia and metabolic acidosis are the most relevant. Goats with PT have a high mortality and their condition can deteriorate very fast. Based on the authors’s experience, a good strategy to minimize the economic losses caused by PT is to focus on the offspring survival rate since an early decision (induction of kidding or caesarian surgery) can increase the number of alive kids.

## Background

Pregnancy toxaemia in small ruminants is a metabolic disease that occurs in pregnant ewes or does, it is caused by negative energy balance and is nearly always associated with rapid growth of multiple foetuses during late gestation [1, 2]. The clinical signs most consistently found are anorexia, ruminal atony, polypnea, drooping ears, a preference for recumbency with reluctance to stand or walk, inability to stand or walk and swelling (subcutaneous edema) of the limbs [1–4]. Less frequently present are nervous signs such as “star gazing”, incoordination, and muscle tremors [1–4]. The major blood chemistry alterations observed in PT goats are increased values of BHBA, hypoglycaemia, hypokalaemia and a marked metabolic acidosis [1–4]. Other metabolic abnormalities may include hypocalcaemia, hypophosphataemia and increased activities of hepatic enzymes in blood serum [5].

Previous studies [5] have shown high case fatality rates in goats affected by PT exceeding 80 % in untreated animals. A different study [4] has shown the case fatality rate to be 86 % (19 of 22) even when kidding was induced or a caesarian surgery was performed and medical treatments (fluids, electrolytes, glucose and propylene glycol) were vigorously administered. Considering this high rate of mortality of goats affected with PT, in this study we have used offspring survival rate when different clinical options are taken as an outcome measure. We hypothesized that the latter would vary depending on clinical signs and clinical pathology parameters of the dam as this focus seems to be a more useful strategy to minimize economical losses.

As the onset of clinical signs in PT affected animals is often difficult to identify due to its subtlety, the course of the disease may progress rapidly after the diagnosis is established. Therefore, clinical decisions regarding treatment and management must be made and implemented rapidly. In a previous study [4], it was found that 58 % (11 of 19) of the PT does died within 24 h after the clinical signs were first observed, and only one lived longer than 3 days. This implies that treatment must be initiated early in the course of the disease, or it will nearly always be unsuccessful [1, 2, 4, 6]. The first decision to be made is whether to simply (1) to attempt to treat the patient, (a) by administering supportive treatments in the interest of addressing the metabolic abnormalities that are present (especially metabolic acidosis, hypokalaemia, and hypoglycaemia) and (b) by terminating the pregnancy or (2) euthanize the patient,. If a decision is made to attempt to treat the patient, the next decision to be made will be selecting the method used for terminating the pregnancy. The two available choices are (1) caesarian surgery or (2) pharmaceutical induction of lambing/kidding. There appears to be an unreported but generalized opinion amongst the veterinary medical profession that caesarian surgery is the best option for saving the life of the dam and kids. However, the cost of this option is higher for the owner than the cost of pharmaceutical induction of parturition.

The clinical signs that have been most frequently found to be associated with a poor prognosis by other authors are (1) recumbency (lateral is more adverse than sternal), (2) drooping ears, and (3) neurologic signs (especially opisthotonus or “star gazing”, incoordination, and muscle tremors) [1–5].

Certain blood parameters have been used as possible prognostic indicators in sheep with PT, including fructosamine [7], alkaline phosphatase [8], BHBA [9] and pH [4]. In a previous study with 22 goats [4], it was shown that blood pH was the best available clinical pathology prognostic indicator: when the blood pH of the dam was below 7.12, the case fatality rate was 100 %. In these cases, euthanasia (or possibly, caesarian surgery) could have been indicated. When the pH was between 7.12 and 7.20, caesarian surgery was performed as soon as possible. Based on what is presently known, when the blood pH is above 7.20, caesarean section appears to have little advantage over pharmacologic induction of kidding, with respect to the survival rates likely to be obtained, and would be expected to be more cost-effective than surgery. These recommendations are based on experiences with a relatively limited number of cases, and are subject to future revisions, as more case studies become available for analysis [4].

The objective of the present study was to establish a prognosis for dairy goats diagnosed with PT, utilizing the blood values for pH, BHBA, glucose, ions (K^+^, Na^+^, Cl^−^), blood gases and clinical signs. It was our intention that these data would enable the construction of a decision-making tree that would allow a more accurate identification which cases should be treated by caesarian surgery, and which can be treated adequately with more conservative and less expensive pharmaceutical methods for terminating pregnancy. As far as we are aware this is the first study that tries to establish a prognosis for PT does based on clinical signs and on haematological parameters, furthermore we provide valuable information on the realistic treatment goals and objectives.

## Methods

### Herd characterization

This study was performed on a 1,800-head dairy goat farm, located 40 km northeast of Lisbon, Portugal. The goats were of two breeds: Alpine (approximately 800 animals) and Saanen (approximately 800 animals), with some animals being crosses of these two breeds (approximately 200 animals). All goats were continuously housed in confinement and all adult lactating does had access to free stalls. There were three kidding seasons per year, beginning in January, April and October. Each kidding season began on the first day of the month, and continued for 45 days. Daily milk production in this herd averaged approximately 3 L/doe. Machine milking was performed twice daily.

### Feeding and herd management

The lactating goats were offered a total mixed ration (TMR) consisting of corn silage, alfalfa hay, brewer’s grains, rye-grass hay and a concentrate mix, fed ad libitum. The percentage of the elements of the TMR varied accordingly to the production level of the goats [high-producing goats (>3 L), low-production goats (<3 L)] (Table 1).

**Table 1:** Proximate analyses of and dry matter intakes obtained with three rations fed to lactating and dry dairy goats

Nutrient	High Producing Does	Low Producing Does	Dry Does
Dry Matter Intake (Kg/day)	2.40–3.00	1.80–2.00	1.40–1.50
Dry Matter (%)	Max: 50	Max: 50	80–90
Crude Protein (% of DM)	17–18	16–17	12
Crude Fat (% of DM)	5	5	4
Crude Fiber (% of DM)	16	17	17
Acid Detergent Fiber (% of DM)	19	20	21
Neutral Detergent Fiber (% of DM)	28	30	31
Starch (% of DM)	15–18	12–14	10–15
Ca (% of DM)	0.80–0.90	0.75–0.80	0.35
P (% of DM)	0.40–0.50	0.35–0.40	0.25
Mg (% of DM)	0.25–0.30	0.25–0.30	0.20
Salt (% of DM)	0.40–0.50	0.40–0.50	0.25

The TMR was distributed once daily. All the adult goats had free access to mineral blocks containing salt, magnesium and trace amounts of iron, iodine, cobalt, manganese, zinc and selenium, in appropriate quantities.

One month before kidding, does producing less than 0.5 L of milk/day were moved to a separate pen (with other dry goats), milking was ceased, the TMR was withdrawn, and wheat straw was fed, ad libitum. In addition, 1 kg/head/day of a different concentrate mix was provided, split into 4 feedings, equally distributed throughout each 24-h period (Table 2). The goats producing more than 0.5 L of mild /day remained in the low production (lactating) goat group and sometimes kidded while still lactating.

**Table 2:** Composition of the commercial concentrate mixes fed to the lactating and dry goats

Ingredient	High and Low Production Does	Dry Does
Ground corn	15 %	17 %
Ground barley	12.5 %	10 %
Corn gluten meal	12 %	15 %
Soybean oil meal	11.5 %	12 %
Canola oil meal	9.5 %	
Dried citrus pulp	7.2 %	1.4 %
Palm oil	7 %	5 %
Wheat bran	6.8 %	13 %
Sunflower oil meal	5.5 %	
Ground wheat	5 %	10 %
Protected fat	2 %	1.8 %
Cane molasses	2 %	3.5 %
Distillers dried grains		6.5 %
Calcium, phosphorus and salt	2 %	2.8 %
Buffers	1.7 %	1.7 %
Micro minerals and vitamins	0.3 %	0.3 %

### Description of the study

The present study began in January 2013, ended in April 2015, spanned eight kidding periods and involved a total of 32 PT goats. Of the 32 PT does, 12 were Alpine, 9 were Saanen and 11 were Saanen-Alpine cross-breds. One PT doe in this study gave birth in January/February 2013, 4 in April/May 2013, 5 in September/October 2013, 5 in January/February 2014, 4 in April/May 2014, 1 in September/October 2014, 4 in January/February 2015 and 8 in April/May 2015. All the goats included in this study were dry (non-lactating) goats.

The prevalence of PT in the periods of the study was 7.1 % (38/352) in January/February 2013, 5.3 % (22/415) in April/May 2013, 2.0 % (7/356) in September/October 2013, 6.9 % (29/418) in January/February 2014, 4.3 % (17/393) in April/May 2014, 1.6 % (6/370) in September/October 2014, 4.7 % (19/405) in January/February 2015 and 4.6 % (19/416) in April/May 2015.

### Inclusion criteria

Any pregnant doe in the dry doe pen that refused to eat when fresh feed (concentrate) was offered (4 times during a 24 h period) was considered to be a PT suspect and her blood BHBA value was determined using an electronic on-farm test^1^. If this value was found to be 3 mmol /L, or greater, she was considered a PT doe and handled accordingly. This inexpensive method of assessing BHBA status has recently been validated [10]. Anorectic goats with BHBA values lower than 3 mmol /L were treated with oral electrolytes (70 % glucose, 15 % sodium chloride and 12 % sodium bicarbonate, dissolved in 1.5 L of water) and propylene glycol (50 ml) and rechecked in a daily basis until they resumed eating or developed a BHBA value in excess of 3.0 mmol/L.

At the time that each PT doe was enrolled in this study, a physical examination was performed and clinical signs (of disease) and vital signs (rectal temperature, heart rate, respiratory rate and ruminal motility) were determined and recorded. In addition, a blood sample was obtained from the jugular vein with a 20-gauge needle using a 1 mL disposable syringe (rinsed with heparin sodium, 5000 IU /ml^2^) and the following parameters were measured on the farm, 2 minutes maximal after blood collection using a portable analyzer^3^: ions (Na^+^, K^+^, Cl^−^ and HCO3^−^), glucose, pH, BE, pCO2 and BUN. Urine was collected from 17 PT goats, when the goat was approached to perform the physical exam and complete urinalyses^4^ were performed.

Of the 32 goats included in the study, kidding was induced in 22 does by intramuscular injection of dexamethasone^5^ (1 mg/10 Kg BW), and dexcloprostenol^6^ (125 μl). A caesarian surgery was performed in the other 10 does.

### Statistical analysis

A two-tailed *t* test for independent samples [11] was used to compare the mean values of the data obtained from goats that died with goats that survived.

The probability of survival of the PT goats was analysed by logistic regression with the PROG LOGISTIC ® from SAS^7^, with a model that included the effect of the analysed parameters (BHBA, pH, Glucose, K^+^, pCO_2_, HCO_3_^−^, BE, Na^+^, Cl^−^ and BUN) individually and as covariables.

The values of these parameters were submitted to an analysis of variance with the PROC GLM ® from SAS with a linear model that only includes the effect of death/survival. After that, the least square means of the different parameters were estimated.

In order to test the association between (1) the occurrence of a clinical signs that were observed in a patient (i.e., polypnea, swollen limbs, anorexia with absence of ruminal motility, recumbency, neurologic signs, and drooped ears) and (2) the death or survival of a patient, (3) number of foetuses alive after inducing the kidding versus caesarian surgery, (4) urine analysis (aciduria) the chi-square test and the Fisher’s Exact Test were used, with the PROC FREQ ® from SAS.

## Results

In the present study, the case fatality rate was 75 % (24/32). Among the goats that were submitted for caesarian surgery, the case fatality rate was 100 % (10/10) and in those where kidding was induced, the case fatality rate was 64 % (14/22).

The 22 goats in which parturition was induced gave birth to a total of 50 kids, 48 were alive (96 %). From these, three goats died before they gave birth. The 10 goats in which a caesarian surgery was performed gave birth to a total of 26 kids, 20 were alive (77 %). This difference is statistically significant (*p* < 0.01).

A comparison of the median ages, BCS, number of fetuses and vital signs of the 24 dead goats and the 8 surviving goats are presented (Table 3). Ruminal motility was significantly more likely to be present in surviving does that in the does that died (*p* < 0.001).

**Table 3:** Ages, BCS, number of Foetuses, vital signs in the 24 dead goats and in the 8 surviving goats

Parameter studied	Dead Goats	Surviving goats	Control goats
	Median (Range)	Median (Range)	(*n* = 22)
Age (years)	4 (2–8)	4 (2–6)	2–6^a^
BCS	3.0 (2–3.5)	3.0 (2.5–3.5)	2.5–2.75^b^
Number of foetuses	3 (2–4)	3 (2–3)	2–3^a^
Rectal Temperature °C)	38.6 (37–39.2)	38.9 (38.7–39.2)	37.7–39.8^a^
Heart rate	114 (60–172)	132 (100–152)	72–154^a^
Respiratory rate	48 (28–100)	48 (32–108)	24–64^a^
	Dead Goats Frequency	Surviving goats Frequency	
Rumen motility *Y* = Yes/*N* = No	Y-4 N-16	Y-5* N-1	Y-1–2 contractions per minute

*BCS* body condition score, *NA* non applicable

^a^Lima et al. [4]

^b^Smith M and Sherman D [1]

*p < 0.001

**Fig. 1:**
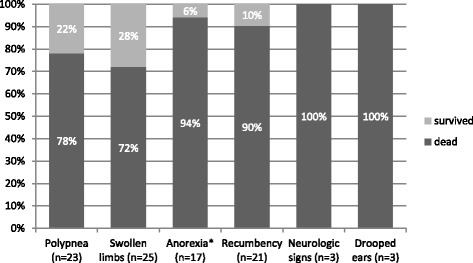
Percentage of the goats that died and survived in function of the most observed clinical signs. The total number of animals that presented each clinical sign is shown

**Fig. 2:**
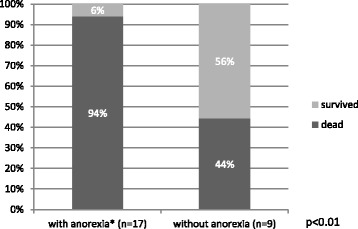
Comparison between the goats that died and survived with and without anorexia. The total number of animals is shown

**Fig. 3:**
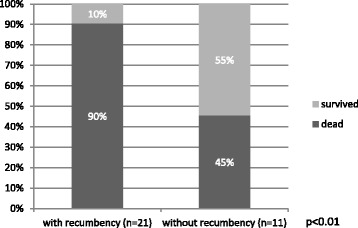
Comparison between the goats that died and survived with and without recumbency. The total number of animals is shown

The following clinical signs were observed in the 32 PT does of this study:

Polypnea (88 %, 28/32), swollen limbs (78 %, 25/32), anorexia with absence of ruminal motility (69 %, 17/26), sternal recumbency, but able to rise upon stimulation (66 %, 21/32), and neurological signs, such as “star gazing” or opisthotonos (9 %, 3/32), and drooping ears (9 %, 3/32). These results are summarized in Fig. 1 and for these there was a statistically significant difference in survival rates in goats with anorexia with absence of ruminal motility (*p* < 0.01) and recumbency (*p* < 0.01) (Figs. 2 and 3, respectively).

Urine samples were collected from 17 PT goats and complete urinalysis performed. Ketonuria and aciduria were the only abnormalities detected and both were present in 13 does, while ketonuria (without aciduria) was present in 4 does. In this study the threshold for aciduria was observed in a goat with a blood pH of 7.35. From the 24 goats that died, urine was collected in 13 and 12 (92 %) of these showed aciduria and ketonuria and one showed only ketonuria (8 %). Urinalysis was performed in four of the eight surviving goats. Three of them showed ketonuria (75 %) and one showed aciduria and ketonuria (25 %). The difference in the occurrence of aciduria between the goats that died and the ones that survived is statistically significant (*p* < 0.05).

**Fig. 4:**
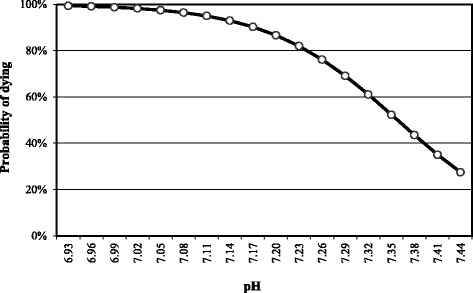
Relationship between the goats’ probability of dying and blood pH

**Fig. 5:**
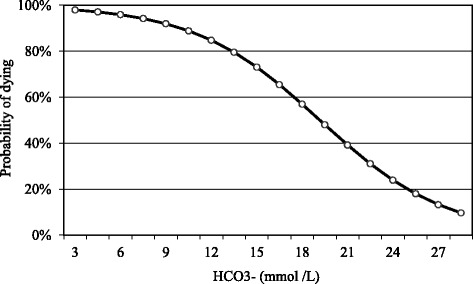
Relationship between the goat’s probability of dying and blood HCO_3_^−^

**Fig. 6:**
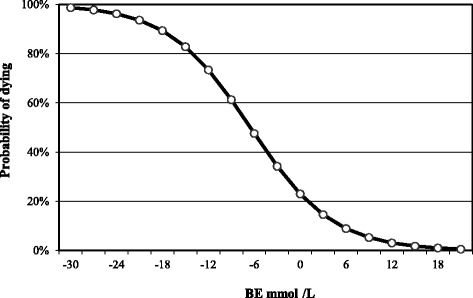
Relationship between the goats’ probability of dying and blood BE

**Fig. 7:**
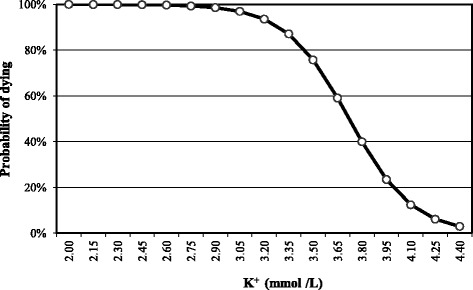
Relationship between the goat’s probability of dying and blood K^+^

**Fig. 8:**
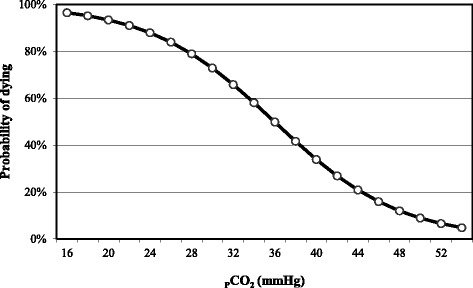
Relationship between the goat’s probability of dying and blood pCO_2_

The blood chemistry values from the 24 dead goats and from the 8 surviving goats are shown in Table 4. Median blood levels of K^+^ (*p* < 0.005), pH (*p* < 0.005), HCO_3_^−^(*p* < 0.001), BE (*p* < 0.001), and pCO_2_ (*p* < 0.005) were significantly lower in the goats that died than in the surviving goats. The probability of survival of the PT goats was analysed by logistic regression and the prognostic values of the blood pH, HCO_3_^−^, BE, K^+^ and pCO_2_ and for the case fatality rate in PT goats are shown (Figs. 4, 5, 6, 7 and 8 respectively).

**Table 4:** Blood chemistry values from the 24 dead goats and from the 8 surviving goats

Parameter Measured	Dead goats Median (Range)	Surviving goats Median (Range)	Control Goats (*n* = 22)
Na^+^ (mmol/L)	139 (123–145	142 (138–145)	128–145^a^
K^+^ (mmol/L	2.7 (1.8–3.8	3.7^*^ (3.5–4.3)	3.3–4.3^a^
Cl^−^(mmol/L)	110 (103–121	109 (96–111)	102–112^a^
Glucose (mmol/L	2.1 (1.1–5.8)	1.9 (1.7–2.8)	2.1–3.4^a^
pH	7.13 (6.84–7.45	7.39^*^ (7.30–7.43)	7.31–7.45^a^
HCO_3_^−^(mmol/L)	8.3 (3.3–21	21^**^ (14–35	19–29^a^
BE (mmol/L)	BE (mmol/L)	−4.5^**^ (−12– + 10)	−0.8– + 4^a^
pCO_2_ (mmHg	26 (16–42)	33^*^ (28–56)	31–44^a^
BUN (mmol/L)	6.1 (2.9–17.6)	4.0 (2.2–7.2)	1–3.8.^a^
BHBA (mmol/ L)	6.5 (3.8–8.6)	6.0 (3.2–10.6)	<0.7^b^

*p < 0.005

**p < 0.001

^a^Lima et al. [4]

^b^Christian JA and Pugh DG [19]

As seen in Fig. 4, at a pH of 6.99, the probability that a PT goat doesn’t survive is 99 %. This probability decreases to 75, 50 and 25 % at values of 7.26, 7.35 and 7.44 respectively. The equation used to build this curve was the following:

$$ P\left(y=1\right)=\frac{e^{87.099-11.837x}}{1+{e}^{87.099-11.837x}} $$

y = probability of dying and x = blood pH

It is possible to observe in Fig. 5 that the probability that a PT goat dies when the blood levels of HCO_3_^−^ are 3 mmol /L is 98 %. This probability decreases to 75, 50 and 25 % at blood levels of 15, 20, 25 mmol /L, respectively. The equation used to build this curve was the following:

$$ P\left(y=1\right)=\frac{e^{4.597-0.240x}}{1+{e}^{4.597-0.240x}} $$

y = probability of dying and x = blood HCO_3_^−^

In Fig. 6, it is possible to observe that when the BE is − 30 mmol/L, the probability that a PT goat dies is 99 %. This probability decreases to 75, 50 and 25 % at values of − 12,−6 and 0 mmol/L respectively. The equation used to build this curve was the following:

$$ P\left(y=1\right)=\frac{e^{-1.207-0.186x}}{1+{e}^{-1.207-0.186x}} $$

y = probability of dying and x = blood BE

When K^+^ is 2 mmol/L or less the probability that the PT goat will not survive is 99 %. This probability decreases to 75, 50 and 25 % at values of ≤ 3.3, ≤ 3.6 and ≤ 4 mmol/L respectively, (Fig. 7). The equation used to build this curve was the following:

$$ P\left(y=1\right)=\frac{e^{11.676+3.213x}}{1+{e}^{11.676+3.213x}} $$

y = probability of dying and x = blood K^+^

When pCO2 is ≤ 18 mmHg, the probability of death of the PT goat is 95 %. This probability decreases to 75, 50 and 25 % at values of ≥ 29, ≥ 36 and ≥ 43 mmHg respectively (Fig. 8). The equation used to build this curve was the following:

$$ P\left(y=1\right)=\frac{e^{-5.962+0.166x}}{1+{e}^{-5.962+0.166x}} $$

y = probability of dying and x = blood pCO_2_

## Discussion

When dealing with a goat with clinical signs of PT in the last days of gestation in practice, a clinician must quickly take some decisions about what should be done. Since only patients in the very early stages of PT respond well to medical treatments [4], the clinician must first decide if the option should be pharmaceutical induction of kidding or caesarian surgery. Goats with PT have a high mortality and their condition can deteriorate quickly [4]. It has been our experience that a good strategy to minimize the economic losses caused by PT is to focus on offspring survival rates rather than on the doe. The criterion used to decide whether to induce parturition or perform a caesarian surgery was the blood pH value, since a decrease in the blood pH has a negative impact on the prognosis of PT goats as it was shown in a previous study [4]. When the pH of the PT goat at the first observation was below 7.15, a caesarian surgery was performed; when the pH was above 7.15, induction of parturition was the option utilized.

It has been reported in sheep that very small changes in maternal pH may cause significant derangement is foetal function and outcome [12]. Since the percentage of the kids alive after caesarian surgery was lower (77 %) than those where parturition was induced (96 %) we hypothesised that the decreased rate of survival of the foetuses is also influenced by the lower blood pH.

Deciding between treatment options in affected animals should be based on the economic value of the mother and offspring, as well as on the clinical signs and certain haematological parameters.

In our study the clinical findings most indicative of a poor prognosis are 1) anorexia with absence of ruminal motility and recumbency (Figs. 2 and 3). We have reported previously [4] and therefore is our experience that drooped ears and nervous signs are associated with a very unfavorable outcome. However in this study there was not a statistically significant association between these two signs and the prognosis. This may be explained by the small size of the sample.

In the present study, the case fatality rate was not influenced by factors such as, age, number of fetuses, BCS, rectal temperature, heart rate or respiratory rate. However, both the surviving and the fatal PT cases had elevated heart and respiratory rates, which could reflect attempts by the animal to compensate for the increased abdominal volume and for the metabolic acidosis that was observed in most of the goats in this study [2, 4, 5].

A recent study [13] tried to identify risk factors for developing PT in goats during the last month of gestation based on their blood BHBA values.

All PT goats in this study had BHBA levels above 3 mmol /L. The difference in BHBA levels (6.5 mmol /L vs 6.0 mmol /L) between the PT goats that died and those that survived was not statistically significant, indicating that BHBA values have little prognostic value for PT in goats.

In this study we have shown statistically significant differences in blood levels of pH, K^+^, HCO_3_^−^, BE, and pCO_2_, between the goats that survived and the ones that died, so these parameters can be used as valid prognostic indicators.

Maternal blood glucose levels can be used as sign of the viability of the foetuses [14–16] Hyperglycaemia in the dam was shown to be associated with fetal death [14–16]. In our study the levels of glycaemia were not elevated in both groups of goats.

It would be useful to establish a correlation between some haematological parameters and clinical signs of PT in goats, using clinical signs such as behavior pattern, ability to stand, nervous signs and drooping ears, as examples. In future studies we will try to explore this area.

During the clinical workup of a PT goat urine analysis is recommended. Unfortunately it is harder to obtain urine samples from does that from cows or ewes. However, a goat, that has been recumbent will often urinate when encouraged to stand [1]. Our results show that the difference in the occurrence of aciduria between the goats that died and the ones that survived is statistically significant (*p* < 0.05) and that goats with ketonuria had a blood BHBA above 3.8 mmol /L. It would be a laudable goal for future work to identify at which levels of BHBA leads to clinically detectable ketonuria in these animals.

According to previous studies, a good predictor of impending death in a PT goat is the blood pH value [4]. However, in our experience, in PT goats, the pH can fall very rapidly, and the physical condition of these goats can also deteriorate very rapidly. Because of this, a timely decision has to be made as to whether to induce kidding with pharmaceuticals or to perform a caesarian surgery. The case fatality rate in these 10 PT does was 100 %. However we were able to remove a total of 26 kids, 20 of which were alive (77 %) after performing the surgery.

Hypokalaemia has been reported as a consistent feature of naturally occurring PT in goats [4, 15] and it can be considered a good indicator of prognosis. As shown in the results the blood levels of K^+^ were significantly lower in the goats that died. It has been shown that in human patients with ketoacidosis and ketonuria, that there is a marked loss of K^+^ in the urine leading to hypokalaemia [17] . It seems logical that hypokalemia observed in PT goats in the present study could have resulted for the same mechanisms that caused hypokalaemia in human patients. On the other hand, goats with PT in the current study had a lower feed intake, which might have contributed to this hypokalaemia.

Due to the fact that mortality in PT goats often exceeds 80 % in untreated animals [5] and that the efficacy of several protocol treatments is not very effective [4] the best strategy to deal with this disease is prevention. However, in our experience it is not very easy to prevent this problem. Goats from the improved breeds Saanen and Alpine have a great prolificacy and tend to accumulate substantial amounts of fat in their abdominal cavity [1, 5]. This reduces space availability for ruminal expansion, therefore these animals are unable to consume sufficient quantities of feedstuffs to satisfy their energy requirements [2]. We have been trying some strategies to decrease the prevalence of PT in this farm.

One strategy is to group goats according to the level of their milk production in order to avoid overfeeding of the lower production goats. The importance of increased exercise to prevent PT in pregnant sheep has been reported [15, 18]. Forced exercise in pregnant sheep was associated with reduced blood concentration of free fatty acids, presumably resulting from increased utilization [18]. In the past we have provided additional pen space to goats in the last month of gestation in order to encourage them to exercise more. The results were not very encouraging.

## Conclusions

The clinical signs most indicative of a poor prognosis are anorexia with absence of ruminal motility and recumbency. Among the blood parameters to be considered, K^+^ and metabolic acidosis are the most relevant. The authors are aware that the findings of this study are based on a relatively small sample size and may be subjected to future revisions, as more case studies become available for analysis. A clinician facing PT in a goat in a commercial farm often faces challenges including availability of proper treatment facilities and the opportunity to perform sequential diagnostic blood testing. However, the clinician still needs to make timely decisions concerning the management of individual PT cases. Because of the high case fatality rate associated with PT, an early decision (induction of kidding or caesarian surgery) is essential to potentially facilitate the production of live kids.

## Abbreviations

BCS: Body condition score
BE: Base excess
BHBA: Beta-hydroxybutyric acid
BUN: Blood urea nitrogen
PT: Pregnancy toxaemia
TMR: Total mixed ration

## Footnotes

1 Precision Xceed, Abbott, UK.

2 B. Braun Medical Lda, Queluz de Baixo).

3 i-Stat, Sensor Devices Incorporated, Waukesha, WI; USA.

4 Aution Sticks, Arkray Europe, Netherlands.

5 Vetacort®, Vetoquinol, Barcarena, Portugal.

6 Gestavet-Prost®, Hipra, Lisboa, Portugal.

7 SAS Institute, Heidelberg, Germany.
